# Identification of the matricellular protein Fibulin-5 as a target molecule of glucokinase-mediated calcineurin/NFAT signaling in pancreatic islets

**DOI:** 10.1038/s41598-017-02535-0

**Published:** 2017-05-24

**Authors:** Tomoko Okuyama, Jun Shirakawa, Hiromi Yanagisawa, Mayu Kyohara, Shunsuke Yamazaki, Kazuki Tajima, Yu Togashi, Yasuo Terauchi

**Affiliations:** 1 Department of Endocrinology and Metabolism, Graduate School of Medicine, Yokohama-City University, Yokohama, Japan; 2 Life Science Center of Tsukuba Advanced Research Alliance, University of Tsukuba, Tsukuba, Japan

## Abstract

Glucokinase-mediated glucose signaling induces insulin secretion, proliferation, and apoptosis in pancreatic β-cells. However, the precise molecular mechanisms underlying these processes are not clearly understood. Here, we demonstrated that glucokinase activation using a glucokinase activator (GKA) significantly upregulated the expression of Fibulin-5 (Fbln5), a matricellular protein involved in matrix-cell signaling, in isolated mouse islets. The islet *Fbln5* expression was induced by ambient glucose in a time- and dose-dependent manner and further enhanced by high-fat diet or the deletion of insulin receptor substrate 2 (IRS-2), whereas the GKA-induced increase in *Fbln5* expression was diminished in *Irs-2*-deficient islets. GKA-induced *Fbln5* upregulation in the islets was blunted by a glucokinase inhibitor, K_ATP_ channel opener, Ca^2+^ channel blocker and calcineurin inhibitor, while it was augmented by harmine, a dual-specificity tyrosine phosphorylation-regulated kinase (DYRK) 1 A inhibitor. Although deletion of *Fbln5* in mice had no significant effects on the glucose tolerance or β-cell functions, adenovirus-mediated *Fbln5* overexpression increased glucose-stimulated insulin secretion in INS-1 rat insulinoma cells. Since the islet Fbln5 expression is regulated through a glucokinase/K_ATP_ channel/calcineurin/nuclear factor of activated T cells (NFAT) pathway crucial for the maintenance of β-cell functions, further investigation of Fbln5 functions in the islets is warranted.

## Introduction

Glucose metabolism plays an important role in normal β-cell functions such as insulin production and insulin secretion, and also in β-cell growth and survival^1, 2^. Glucose signaling in the pancreatic β-cells has also been shown to be involved in β-cell proliferation in both humans and rodents^3–6^. Glucokinase, a member of the hexokinase family, is the predominant enzyme catalyzing the phosphorylation of glucose in the pancreatic β-cells and the liver. Glucokinase acts as a glucose sensor for insulin secretion from the pancreatic β-cells^7^ and is required for the effects of glucose signaling on β-cell proliferation^8^. Heterozygous inactivating mutations of glucokinase cause type 2 maturity onset diabetes of the young (MODY2), and homozygous or compound heterozygous inactivating glucokinase mutations cause a more severe phenotype known as permanent neonatal diabetes mellitus (PNDM), which manifests at birth^9^. On the other hand, heterozygous activating glucokinase mutations cause persistent hyperinsulinemic hypoglycemia (PHHI)^10^, associated with increased β-cell mass and β-cell proliferation^11^. We have shown previously that glucokinase activation ameliorates endoplasmic reticulum (ER) stress-mediated apoptosis of the pancreatic β-cells^12^, while another report revealed that genetic activation of β-cell glucokinase causes cell apoptosis associated with DNA double-strand breaks and activation of the tumor suppressor protein p53^13^. Thus, glucokinase appears to play important roles in β-cell function, replication, and survival. These findings inspired the development of a therapeutic strategy for diabetes by targeting glucokinase. Glucokinase activators (GKAs) increase the glucose affinity and maximum velocity (Vmax) of glucokinase, leading to enhanced glucose-induced insulin secretion from the islets and enhanced hepatic glucose uptake^14^. This ability suggests a potential pharmacological role of GKAs in the treatment of diabetes. However, further investigation is needed to determine the efficacy and safety of GKAs; for example, downstream targets of glucose metabolism in the β-cells have not yet been clearly revealed.

Fibulin-5 (Fbln5; also known as EVEC or DANCE), a matricellular protein, is essential for elastic fiber assembly^15, 16^. Fbln5 is secreted by various cell types, including vascular smooth muscle cells (SMCs), fibroblasts and endothelial cells. Fbln5 expression is usually downregulated after birth, but reactivated upon tissue injury^17, 18^. Fbln5 has several non-elastogenic functions, for example, regulation of proteases via its integrin-binding domain^19–22^. Fbln5 has also been shown to bind to the α5β1 fibronectin receptor and the β1 integrin^21, 23^. Indeed, Fbln5 plays critical roles in cell proliferation, migration and invasion of certain tumors and smooth muscle cells^24, 25^. Mice lacking in Fbln5 exhibit systemic elastic fiber defects, including loose skin, tortuous aorta, emphysematous lungs, and genital prolapse^16, 26^. However, the precise nature of the involvement of Fbln5 in metabolism remains unknown.

In this study, we found that treatment with a GKA induced *Fbln5* gene expression in mouse pancreatic islets. Although it has been reported that interaction of the islets with some specific extracellular matrix molecules is important for islet/β-cell survival^27, 28^, the precise expression levels and roles of these molecules in the pancreatic islets and β-cell functions remain obscure. In this study, we focused on the regulation of *Fbln5* expression in the pancreatic β-cells.

## Results

### Glucokinase activation induced *Fbln5* expression in the pancreatic islets

**Figure 1:**
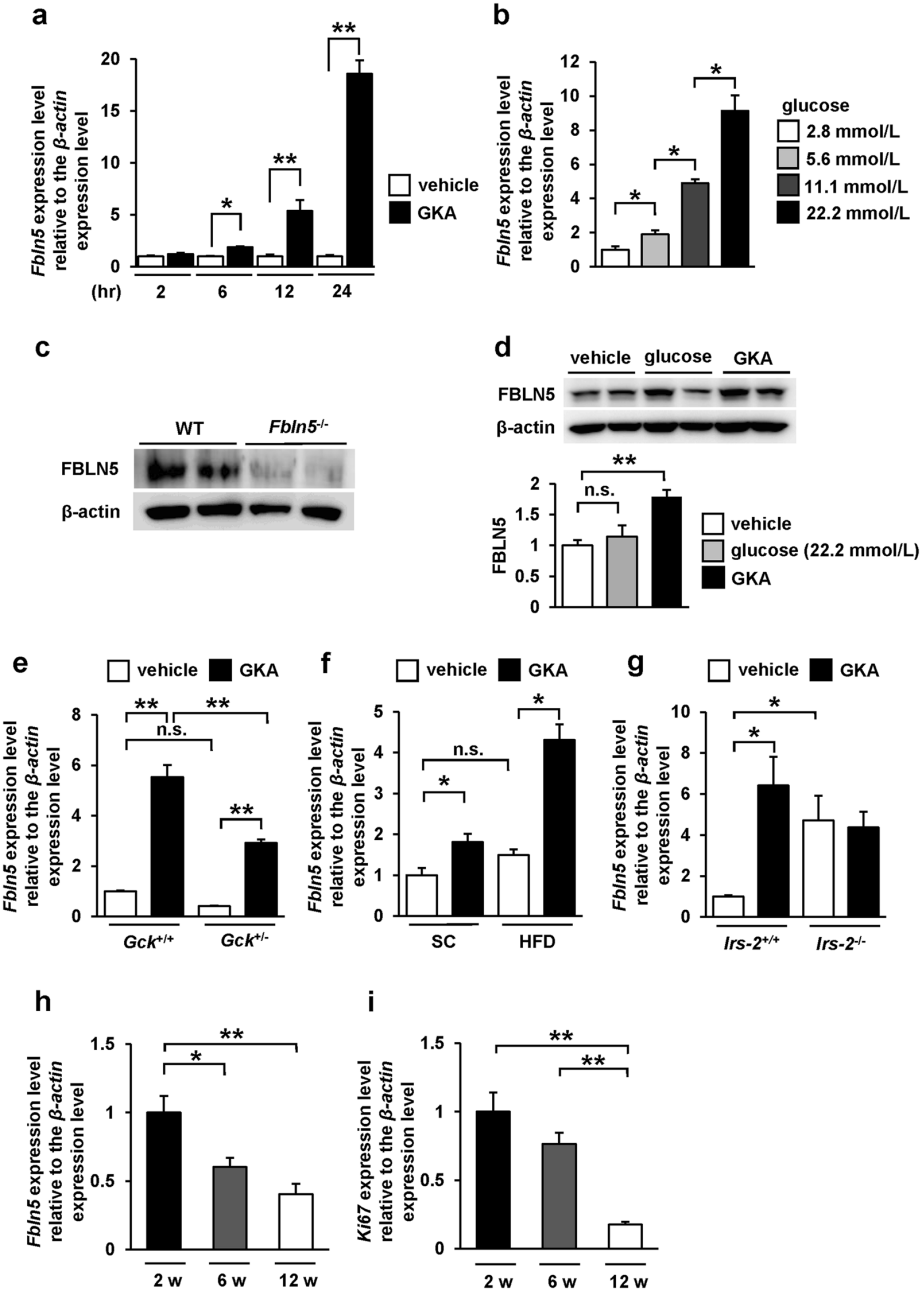
Glucokinase activation was associated with upregulation of *Fbln5* gene expression in pancreatic islets. (**a**) *Fbln5* mRNA expression levels in isolated islets from C57BL/6 J mice incubated for 2, 6, 12 and 24 hours with 30 μmol/L of GKA CpdA or vehicle (DMSO) (n = 4). (**b**) *Fbln5* mRNA expression levels in isolated islets from C57BL/6 J mice (n = 4) after 24 hours’ incubation with 2.8, 5.6, 11.1 or 22.2 mmol/L of glucose. (**c**) Immunoblotting for FBLN5 in islets isolated from wild-type or *Fbln5* ^−/−^ mice. Full-length blots are presented in Supplementary Fig. 4. (**d**) Immunoblotting for FBLN5 in INS-1 cells treated with vehicle, 22.2 mmol/L of glucose, or 30 μmol/L of GKA CpdA for 24 hours (n = 4). Full-length blots are presented in Supplementary Fig. 4. (**e**) *Fbln5* mRNA expression levels in islets from 12-week-old *Gck* ^+/−^ mice and their wild-type littermates after incubation with 30 μmol/L of GKA CpdA for 24 hours (n = 4). (**f**) *Fbln5* gene expression levels in islets isolated from 12-week old standard chow-fed mice (SC) and 20-week of high-fat diet (HFD)-fed mice incubated with or without 30 μmol/L of GKA CpdA (vehicle; DMSO) for 24 hours (n = 4). (**g**) *Fbln5* mRNA expression levels in islets from 12-week-old *Irs-2* ^−/−^ mice and their wild-type littermates after incubation with 30 μmol/L of GKA CpdA for 24 hours (n = 4). (**h**–**i**) mRNA expression levels of *Fbln5* (**h**) or *Ki67* (**i**) in isolated islets from 2-, 6- and 12-week-old C57BL/6 J mice (n = 3–4). Data are represented as means ± SEM. *p < 0.05, **p < 0.01.

At first, we identified by gene expression microarray analysis (GSE41248), that stimulation of mouse pancreatic islets with a GKA for 24 hours induced *Fbln5* expression in the islets (12.6-fold enhanced expression as compared to that in the vehicle control; *p* = 0.0043)^12^. To validate this upregulation of *Fbln5* expression by treatment with a GKA in mouse pancreatic islets, we investigated *Fbln5* mRNA expression in isolated islets from C57BL/6 J mice. Consistent with the results of the microarray analysis, the *Fbln5* mRNA expression in the isolated islets was significantly increased, in a time-dependent manner, by treatment with a GKA (Fig. 1a). Ambient glucose also induced *Fbln5* expression in the islets in a concentration-dependent manner (Fig. 1b). We detected FBLN5 protein expression in the wild-type mouse islets, as well as in INS-1 rat insulinoma cell line (Fig. 1c and d) but not in the *Fbln5*-deficient (*Fbln5* ^−/−^) islets (Fig. 1c). The treatment with a GKA also increased FBLN5 protein expression levels in INS-1 cells (Fig. 1d). Moreover, in glucokinase hetero-deficient (*Gck* ^+/−^) mouse islets, GKA-stimulated *Fbln5* mRNA expression levels were reduced as compared to those in the islets from wild-type mice (Fig. 1e). No difference was detected in *Fbln5* mRNA expression levels between vehicle-treated *Gck* ^+/−^ islets and the wild-type islets (*p* = 0.357) (Fig. 1e). These results suggest that *Fbln5* expression is induced by glucokinase activation in the pancreatic islets. Furthermore, the GKA-induced increase in *Fbln5* expression was more pronounced in the islets of mice reared on a high-fat diet for 20 weeks than in the islets of standard chow-fed mice (Fig. 1f), although there were no significant differences between the vehicle-treated islets from standard chow-fed and high-fat diet-fed mice (*p* = 0.24), consistent with the report that glucokinase-mediated signaling in the β-cells is activated by a high-fat diet^8, 29^. In contrast, in insulin receptor substrate 2 (IRS-2)-deficient (*Irs-2* ^−/−^) mouse islets, basal *Fbln5* expression was significantly increased compared with those of wild-type mice (Fig. 1g). However, the response of *Fbln5* induction to GKA was almost abolished in *Irs-2* ^−/−^ mouse islets (Fig. 1g). It may also explain the more pronounced upregulation of islet *Fbln5* expression in high-fat diet-fed mice than in normal chow-fed mice, as GKA is known to induce IRS-2 expression in the β-cells of mice reared on a high-fat diet^8^. The lack of *Fbln5* induction in *Irs-2* ^−/−^ islets suggests that IRS-2 is involved in the GKA-induced upregulation of islet *Fbln5* expression. Moreover, we found that *Fbln5* was strongly expressed in the islets of 2-week-old pre-weaning mice, the expression level decreasing by 6 or 12 weeks of age (Fig. 1h). This expression pattern of *Fbln5* is consistent with the expression of the proliferation marker Ki67 in the islets (Fig. 1i).

### Glucokinase/K_ATP_ channel/calcineurin/NFAT signaling is required for glucose-mediated *Fbln5* expression in islets

**Figure 2:**
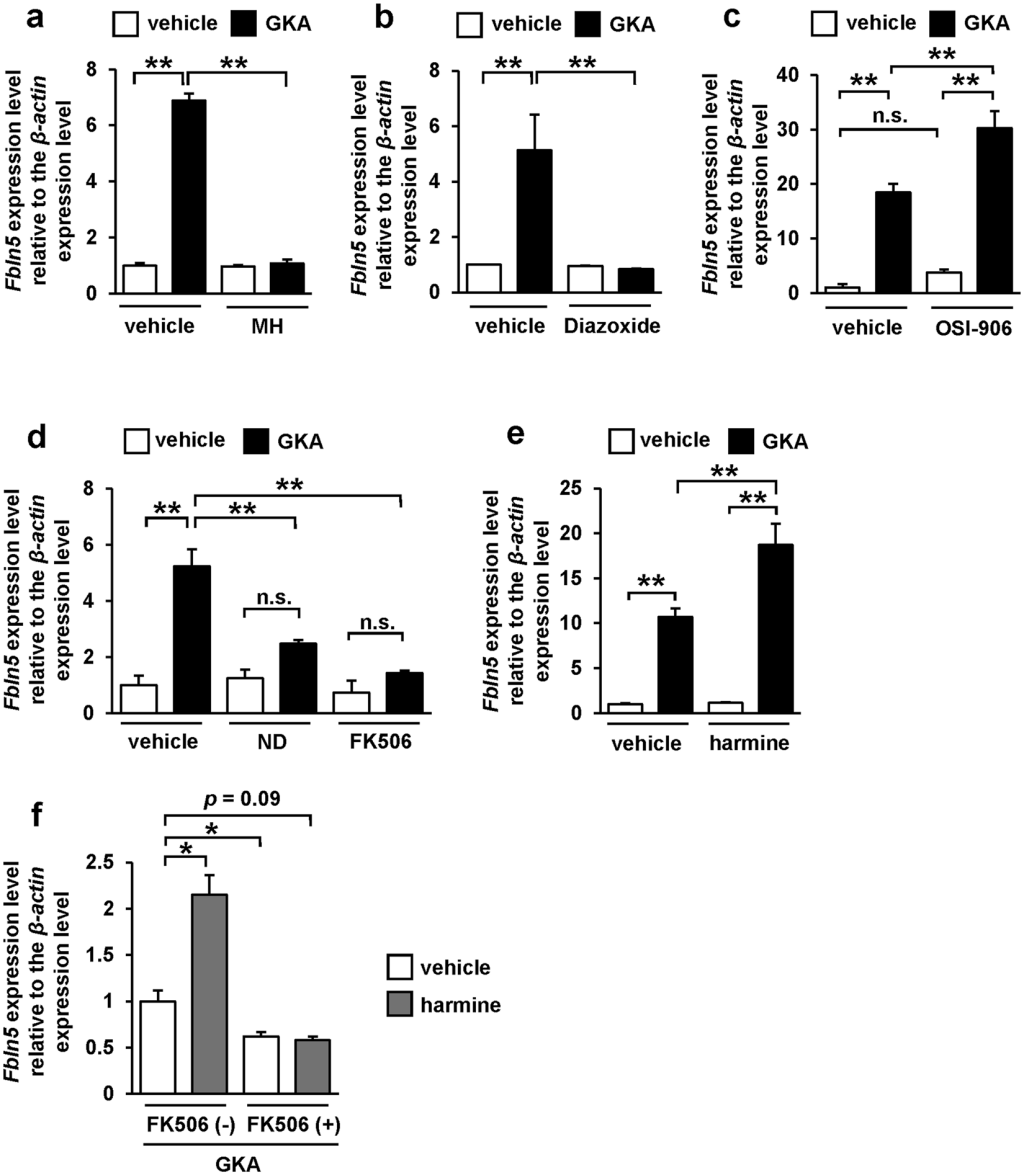
Glucose-signal induced *Fbln5* upregulation via the glucokinase/K_ATP_ channel/calcineurin/NFAT signaling pathway in pancreatic islets. (**a**–**f**) *Fbln5* mRNA expression. Islets from C57BL/6 J mice were incubated with 10 nmol/L of D-mannoheptulose (MH) (n = 4) (**a**), 200 μmol/L of diazoxide (n = 4) (**b**), 200 nmol/L of OSI-906 (n = 4) (**c**), 50 μmol/L of nifedipine (ND), 10 μmol/L of FK506 (n = 3) (**d**), 10 μmol/L of harmine (n = 3) (**e**), or a combination of 10 μmol/L of FK506 and 10 μmol/L of harmine (n = 4) (**f**) for 24 hours in the presence or absence of 30 μmol/L of GKA CpdA (vehicle; DMSO). Data are represented as means ± SEM. *p < 0.05, **p < 0.01.

We next assessed the signaling pathways underlying the GKA-induced upregulation of *Fbln5* in the pancreatic islets. Treatment with D-mannoheptulose, a specific inhibitor of glucokinase, completely abolished the GKA-induced upregulation of *Fbln5* in the pancreatic islets (Fig. 2a). In addition, treatment with diazoxide, a K_ATP_ channel (ATP-sensitive potassium channel) opener, also suppressed the GKA-induced elevation of *Fbln5* expression in the islets (Fig. 2b). Treatment with OSI-906, a dual insulin and IGF-1 receptor inhibitor, did not reduce the *Fbln5* induction by GKA, but enhanced it (Fig. 2c). These results imply that an influx of Ca^2+^ into the β-cells via depolarization of the plasma membrane accompanied by the closure of K_ATP_ channel, and not the autocrine action of insulin, is involved in the GKA-induced upregulation of *Fbln5* in the pancreatic islets.

Calcineurin is activated in an intracellular Ca^2+^-dependent manner^30^, leading to NFAT activation by dephosphorylation and subsequent translocation of NFAT from the cytosol to the nucleus^31^. Glucose-induced regulation of *Irs-2* expression has been reported to be mediated via this Ca^2+^/calcineurin/NFAT signaling in the pancreatic β-cells^32^. Hence, we evaluated the effects of a Ca^2+^ channel blocker, a calcineurin inhibitor, and a DYRK1A inhibitor on the upregulation of *Fbln5* in the islets treated with a GKA. Blockade of the L-type voltage-dependent Ca^2+^ channels (L-type VDCCs) with nifedipine in isolated mouse islets abrogated the GKA-induced increase in *Fbln5* expression in the islets (Fig. 2d). Moreover, treatment with FK506, which specifically inhibits calcineurin activity, also almost completely abolished the GKA-induced increase in *Fbln5* expression in the pancreatic islets (Fig. 2d). Dual-specificity tyrosine phosphorylation-regulated kinases (DYRKs), including DYRK1A, inactivate the NFAT1 proteins by phosphorylating its SP-3 motif^33^. Notably, harmine, a DYRK1A inhibitor, enhanced the *Fbln5* expression induced by treatment with a GKA (Fig. 2e). The effect of harmine on the increment in *Fbln5* expression in islets was blunted in the presence of FK506 (Fig. 2f). These results suggest that the transcriptional regulation of *Fbln5* in the islets is mediated by glucose signaling and downstream Ca^2+^/calcineurin/NFAT signaling.

### *Fbln5*^*−/−*^ mice exhibited normal glucose tolerance and normal glucose-stimulated insulin secretion and β-cell proliferation evoked by GKA

**Figure 3:**
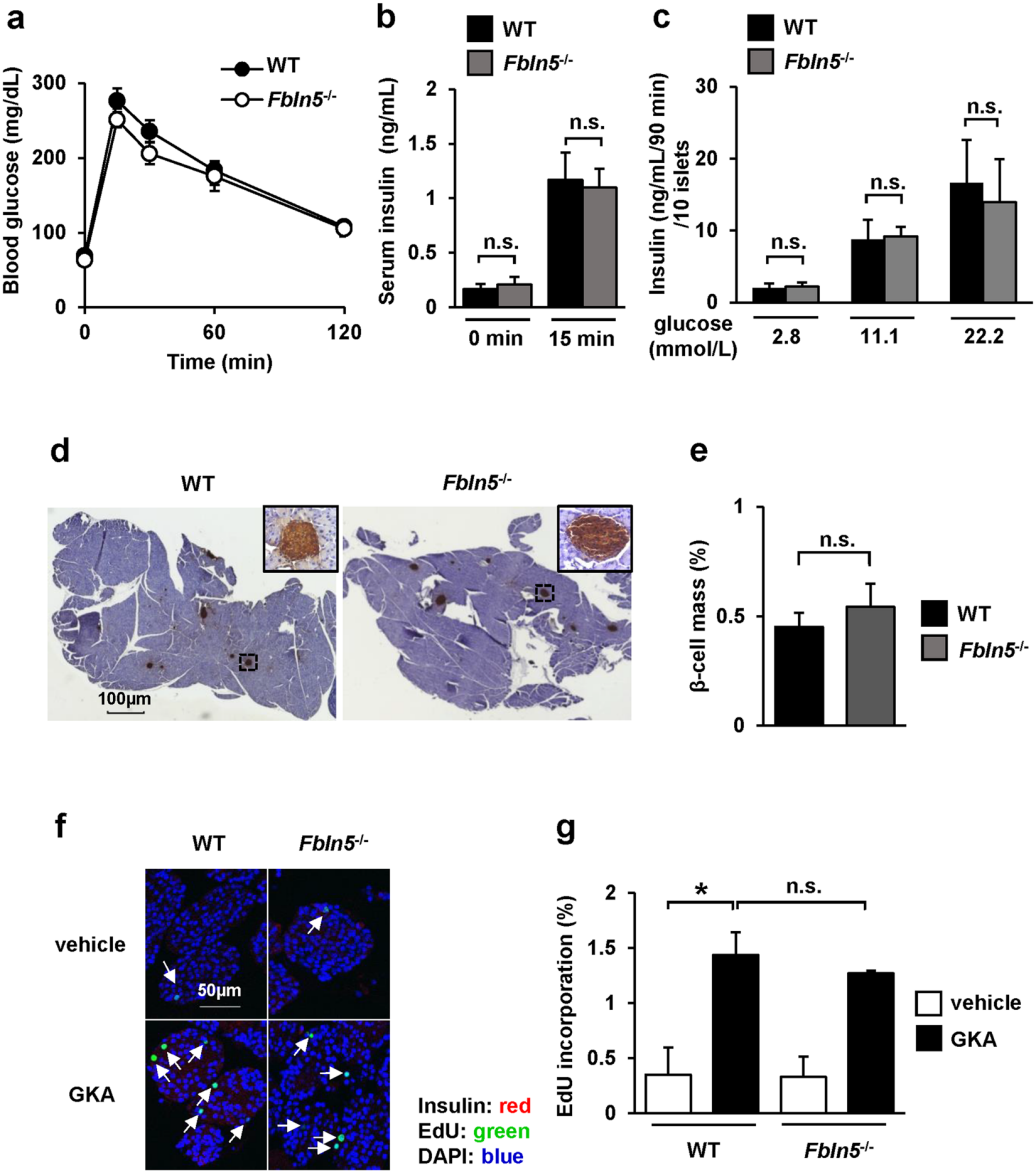
*Fbln5* deficiency in mice had no effect on glucose tolerance, glucose-stimulated insulin secretion or β-cell proliferation. (**a**) Blood glucose levels during an oral glucose tolerance test (OGTT) in 8-week-old *Fbln5* ^−/−^ mice and wild-type mice (1.5 g/kg body weight, n = 11–13). (**b**) Serum insulin levels during the OGTT (n = 11–13). (**c**) Glucose-stimulated insulin secretion in ten isolated islets from 8-week-old *Fbln5* ^−/−^ mice and wild-type mice (n = 6–12). (**d**,**e**) β-cell mass in 8-week-old *Fbln5* ^−/−^ mice and wild-type mice (n = 5). Representative images of the pancreas showing brown staining of insulin (**d**) and the ratio of the β-cell mass relative to the area of the whole pancreas (**e**) (n = 5). The scale bar represents 100 µm in the pancreas images. (**f**,**g**) EdU incorporation in islets isolated from 8-week-old C57BL/6 J mice. Representative images of pancreatic islets after 48 hours of incubation with EdU in the presence or absence of 30 μmol/L of GKA CpdA (**f**). Insulin is stained red, nuclei are stained blue with 4′, 6-diamidino-2-phenylindole [DAPI], and EdU-positive nuclei are stained green. The white arrows indicate EdU-positive β-cells. The scale bar represents 50 µm. The ratio of EdU-positive β-cells to the total count of insulin-positive β-cells is shown (**g**). More than 3000 cells were counted in each group (n = 2). Data are represented as means ± SEM. *p < 0.05.

To investigate the role of Fbln5 in glucose metabolism and insulin secretion, we used 8- to 12-week-old *Fbln5* knockout (*Fbln5* ^−/−^) mice^16^ to evaluate whether *Fbln5* deletion may influence glucose homeostasis *in vivo*. *Fbln5* ^−/−^ mice showed normal glucose tolerance and comparable insulin secretion during an oral glucose tolerance test (Fig. 3a and b). No significant difference in glucose-stimulated insulin secretion was observed between islets isolated from *Fbln5* ^−/−^ mice and wild-type mice (Fig. 3c). These results imply that Fbln5 has no effect on insulin secretion in healthy young adult mice.

We next assessed the β-cell mass in the 8- to 12-week-old *Fbln5* ^−/−^ mice. No significant differences in the islet morphology and the β-cells area relative to the total pancreatic area were observed between the wild-type mice and *Fbln5* ^−/−^ mice (Fig. 3d and e). Furthermore, we evaluated the GKA-induced β-cell proliferation activity in *Fbln5* ^−/−^ and wild-type islets. Treatment with GKA for 48 hours markedly increased the EdU-incorporated proliferating insulin-positive β-cells to a similar extent in the islets isolated from both genotypes of mice (Fig. 3f and g). On the other hand, the fluorescent intensity of insulin was significantly increased in the GKA-treated islets compared with the vehicle-treated islets in wild-type mice, but not in *Fbln5* ^−/−^ mice (see Supplementary Fig. S1a). This result in islets from wild-type mice is consistent with the observation that glucokinase activation enhances insulin gene expression and insulin secretion in β-cells^12, 34^. However, GKA-induced insulin secretion was not decreased in *Fbln5* ^−/−^ islets compared with wild-type islets (see Supplementary Fig. S1b). Insulin content in GKA-treated *Fbln5* ^−/−^ islets showed a tendency to be decreased compared to wild-type islets, but it did not reach statistical significance (see Supplementary Fig. S1b). Thus, Fbln5 is not required for the development and maintenance of β-cell function or proliferation.

In immunohistochemical analysis of paraffin-embedded endocrine pancreatic tissue from 8-week-old wild-type mice and *Fbln5* ^−/−^ mice, FBLN5 was seemed to be around CD34 (endothelial marker) -positive interstitial tissue, but not in β-cells, α-cells, or δ-cells in the islets (see Supplementary Fig. S2). Next, fetal pancreatic tissue paraffin sections from wild-type mice at the age of embryonic day 15 were immunostained for FBLN5. The area and intensity of the FBLN5 signal in the islets seemed to be more abundant compared with those in adult mice (see Supplementary Fig. S3a). Then, we used non-paraffinized cultured islets from 8-week-old wild-type mice for immunostaining. Notably, FBLN5-positive β-cells were detectable in non-paraffinized adult wild-type islets (see Supplementary Fig. S3b). In addition, FBLN5 is observed at cytoplasmic granular structures in INS-1 cells, as shown in Supplementary Fig. S3c, suggesting that FBLN5 is also expressed in β-cells.

### Adenovirus-mediated *Fbln5* overexpression increased glucose-stimulated insulin secretion and inhibited cell proliferation in INS-1 cells

**Figure 4:**
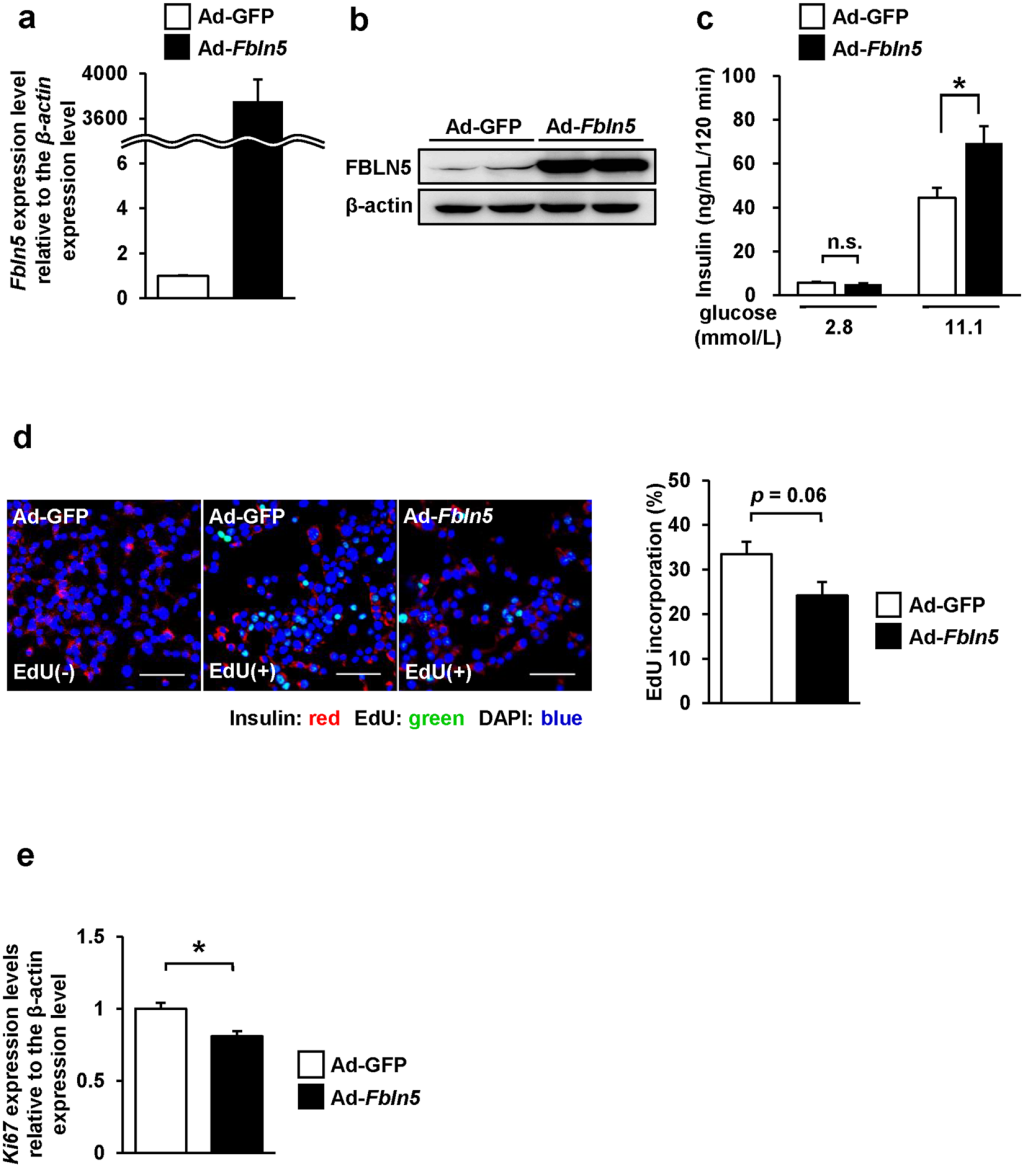
*Fbln5* overexpression in INS-1 cells enhanced glucose-stimulated insulin secretion, but inhibited β-cell proliferation. (**a**) *Fbln5* mRNA levels in INS-1 cells after infection with Ad-GFP or Ad-*Fbln5* for 48 hours (n = 2). (**b**) Immunoblotting for FBLN5 in INS-1 cells infected with Ad-GFP or Ad-*Fbln5*. Full-length blots are presented in Supplementary Fig. 4. (**c**) Glucose-stimulated insulin secretion by INS-1 cells infected with Ad-GFP or Ad-*Fbln5* (n = 6–12). (**d**) EdU incorporation in INS-1 cells infected with Ad-GFP or Ad-*Fbln5* for 48 hours. Cells were incubated for 3 hours with 10 µM of EdU after adenovirus infection. *left:* Representative images of Ad-GFP infected INS-1 cells or Ad-*Fbln5* infected INS-1 cells stained for EdU. Ad-GFP infected cells incubated without EdU are shown as control. Insulin is stained red, nuclei are stained blue with DAPI, and EdU-positive nuclei are stained green. The scale bar represents 50 µm. *right:* The ratio of EdU-positive cells to the total count of insulin-positive cells is shown. More than 6000 cells were counted in each group (n = 4). (**e**) *Ki67* mRNA expression in INS-1 cells infected with Ad-*Fbln5* or Ad-GFP for 48 hours (n = 4). Data are represented as means ± SEM. *p < 0.05.

Next, we evaluated the properties of Fbln5 by forced expression of *Fbln5* gene expression using an adenovirus vector (Ad-*Fbln5*) in INS-1 cells. Following adenovirus-mediated infection of Ad-*Fbln5* in INS-1 cells, overexpression of *Fbln5* was confirmed by measuring the mRNA and protein expression levels (Fig. 4a and b). The cells overexpressing *Fbln5* showed enhanced insulin secretion in the presence of 11.1 mmol/L glucose as compared to the control cells (1.6-fold, *p* = 0.017), although basal insulin secretion was not significantly different between the Ad-*Fbln5*- and Ad-GFP- infected INS-1 cells (Fig. 4c). The effects of *Fbln5* overexpression on the cell proliferation activity was evaluated by measuring the EdU incorporation and *Ki67* expression in Ad-*Fbln5*-infected INS-1 cells. We were almost able to ignore the GFP-signals when we adjusted the gain of signals according to the fluorescent intensity of EdU. (Fig. 4d left panel). The ratio of EdU-incorporated proliferating INS-1 cells to the total count of INS-1 cells tended to be decreased in the Ad-*Fbln5*-infected cells as compared with that in the control cells (Fig. 4d). In addition, Ad-*Fbln5*-infected INS-1 cells showed significant reduction in the *Ki67* expression (Fig. 4e). These results indicate that overexpression of *Fbln5* enhances insulin secretion whereas decreases cell proliferation in β-cells.

## Discussion

**Figure 5:**
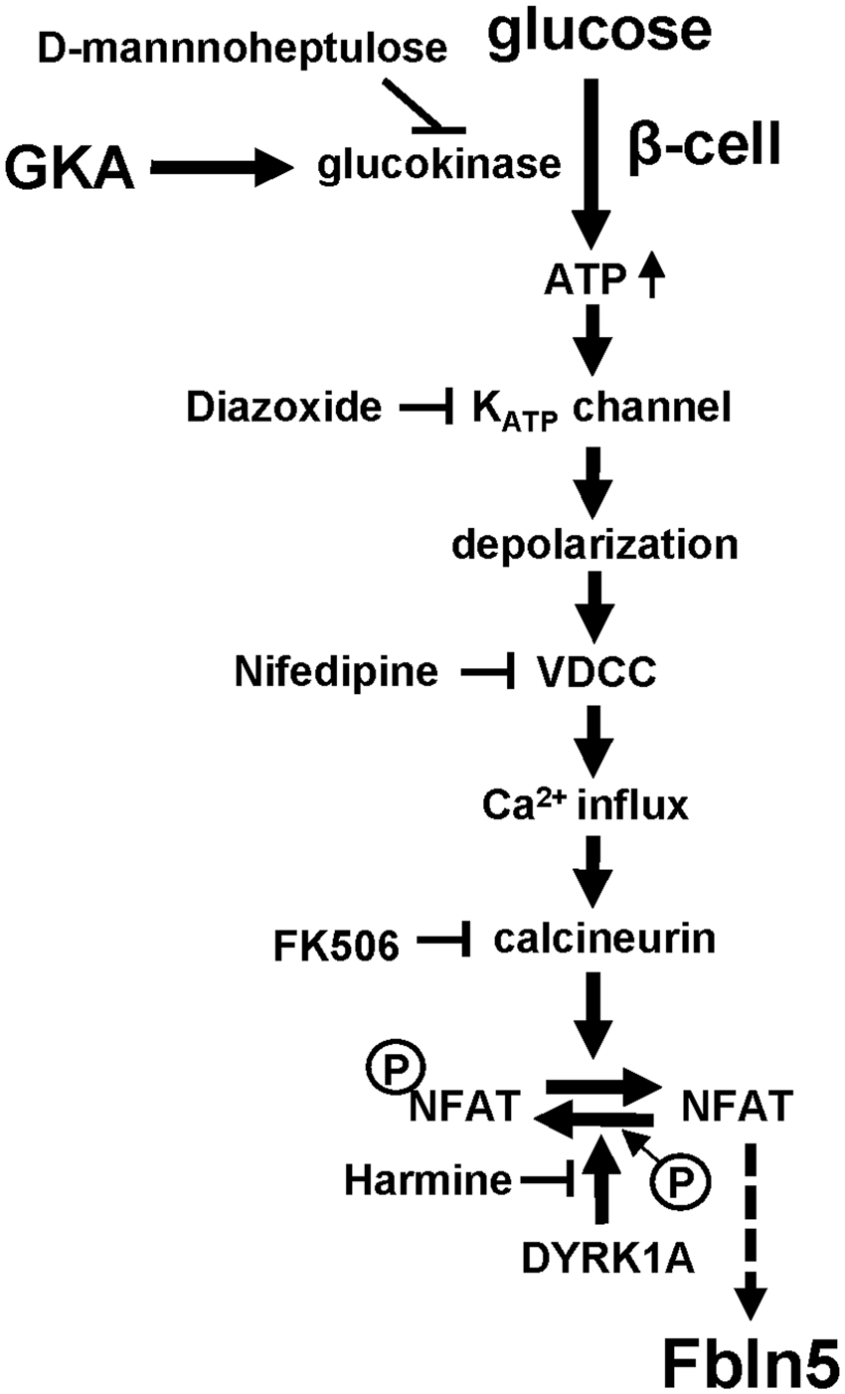
A schematic representation of glucokinase-mediated *Fbln5* expression in pancreatic islets. *Fbln5* gene expression was induced by glucokinase activation through ambient high glucose concentrations or GKA in pancreatic islets. Depolarization of the membrane accompanied by the closure of K_ATP_ channels, Ca^2+^ influx and calcineurin activation are required for this *Fbln5* upregulation. A DYRK1A inhibitor, harmine, enhanced *Fbln5* expression in the islets induced by glucokinase activation, possibly via NFAT signaling. DYRK1A; dual-specificity tyrosine phosphorylation-regulated kinase 1 A, GKA; glucokinase activator, K_ATP_ channel; ATP-sensitive potassium channel, NFAT; nuclear factor of activated T cells, VDCC; voltage-dependent Ca^2+^ channels.

In this study, we showed the glucose signaling-induced transcriptional regulation of Fbln5 in pancreatic islets, which is mediated by glucose metabolism via glucokinase and downstream Ca^2+^/calcineurin/NFAT signaling pathway (Fig. 5).

Because β-cells are exposed to high ambient glucose concentrations under the diabetic condition, glucokinase, which acts as a glucose sensor, transmits the impact of the hyperglycemia to the β-cells. In the pancreatic islets, glucokinase is mainly expressed in the β-cells, with very low levels of expression observed in the α-cells (unpublished data). We confirmed GKA-induced increase in FBLN5 expression in INS-1 cells. Immunohistochemical analysis of INS-1 cells also supported that FBLN5 is expressed in the β-cells. However, further investigation using more specific antibody is needed to clarify the localization of FBLN5 since we observed FBLN5 signal not only in cytoplasm but also in the nucleus in INS-1 cells. FBLN5 immunofluorescence from paraffin-embedded tissue was mainly detected in non-β-cells tissue in the islets. FBLN5 is reported to be deposited on microfibrils during the development of mature elastic fiber^26^. Co-staining FBLN5 with CD34 in islets from paraffin-embedded specimens indicated that FBLN5 is strongly expressed in endothelial cells or small vessels in islets, consistent with the previous reports, which showed FBLN5 secretion from vascular smooth muscle cells or endothelial cells^35, 36^. FBLN5 was also detectable in β-cells in the non-paraffinized cultured islets. It is therefore possible that FBLN5 in β-cells was lost or masked in the process of paraffin embedding or deparaffinization. Since dual inhibition of insulin and IGF-1 receptor with OSI-906 did not abrogate GKA-induced *Fbln5* upregulation in the islets, it is unlikely that this upregulation is mediated by an autocrine action of insulin on the insulin receptor. Because FBLN5 is a secreted protein, islet-derived FBLN5 might be deposited outside of the islets and play a role in non-islet tissue functions.

Our data showed that GKA-induced upregulation of *Fbln5* was more pronounced in islets isolated from obese mice reared on a high-fat diet than in the islets of control mice fed normal chow. This could be explained by the involvement of glucokinase in the compensatory β-cell hyperplasia induced by a high-fat diet^8, 29^. Consistent with this notion, we found that *Irs-2* deletion increased basal *Fbln5* expression and attenuated the GKA-induced upregulation of *Fbln5* in the isolated islets. Chronic hyperglycemia in *Irs-2* ^−/−^ mouse may cause the elevation in *Fbln5* in the islets at the basal state. Other factors that are related to insulin resistance with *Irs-2* deletion in mice can possibly be involved in this basal elevation. Glucose-induced transcriptional regulation of *Irs-2* gene expression in the β-cells is mediated by the Ca^2+^/calcineurin/NFAT pathway^32^, which is involved in β-cell proliferation in mice and humans^37, 38^. In addition, the DYRK1A inhibitor has been demonstrated to enhance β-cell proliferation in mice^39, 40^. It is also reported that GKA-induced increase of the mRNA expressions of *Nfatc1* and its downstream genes are involved in β-cell maturation and β-cell proliferation in neonatal islets^38^. A recent study showed that glucose-induced mouse pancreatic β-cell proliferation is mediated via IRS-2, MTOR and cyclin D2, but not by the insulin receptor^41^. We also found that the *Fbln5* expression was higher in the islets harvested from pre-weaning mice, which showed robust β-cell proliferation as confirmed by the high *Ki67* expression. *Fbln5* is strongly expressed during embryogenesis and plays a role in tissue remodeling^25^. Therefore, *Fbln5* could be a predictor for compensatory β-cell proliferation and remodeling of β-cell mass induced by activation of IRS-2 expression.

How does NFAT signaling regulate the transcriptional activity of *Fbln5* ? *Fbln5* expression is positively regulated via transforming growth factor β1 (TGF-β1) in fibroblasts or epithelial cells^25^. Calcineurin inhibitors induce the TGF-β receptor-triggered signaling cascade in the mesangial cells or kidney^42^. Hypoxia-inducible factor-1α (HIF-1α) is also a Fbln5-inducible factor in the endothelial cells^43^. HIF-1α expression is also reportedly regulated through calcineurin activity or dephosphorylation of RACK1 in mast cells^44, 45^. We have identified NFAT consensus sequences in the 5-upstream region of the mouse *Fbln5* gene at: −698 to −693 (AGGAAA), +386 to +391 (TGGAAA), +428 to +433 (TGGAAA), +591 to +596 (TGGAAA), and 4 other sites from the first transcription initiation site. Further analysis, including of the TGF-β and HIF-1α pathways, are needed to clarify the precise mechanism of *Fbln5* transcription via NFAT in pancreatic islets.

Loss of systemic *Fbln5* expression had no significant effects on the insulin secretion from the pancreatic islets or β-cell proliferation/expansion in young adult mice, suggesting that Fbln5 does not seem to be involved in β-cell development or functions at this stage of life. However, the effects of Fbln5 on pancreatic β-cell functions under diabetic- or insulin-resistant conditions remain unclear. In addition, we showed that *Fbln5* expression is abundant in the islets from fetal or pre-weaning mice. Testing juvenile mice, therefore, is required for further investigation into the physiological role of Fbln5 in the context of developmental process. *Fbln5* overexpression in INS-1 cells revealed that Fbln5 could positively regulate glucose-stimulated insulin secretion from the pancreatic β-cells. By contrast, *Fbln5* overexpression possibly suppress cell proliferation in the INS-1 cells. In fact, Fbln5 overexpression decreased *Ki67* expression in INS-1 cells, although *Fbln5* and *Ki67* expression were increased in proliferating juvenile islets. Fbln5 is reported to promote cell proliferation or tumor growth in mouse 3T3-L1 fibroblasts or human HT1080 fibrosarcoma cells^25^, mouse pancreatic ductal adenocarcinoma^46^, and human gastric cancer MGC-803 cells^47^. On the other hand, several previous studies have demonstrated inhibition of cell proliferation by *Fbln5* overexpression in mouse vascular smooth muscle cells^18^, human breast cancer cells^48^, mink lu Mv1Lu epithelial cells^25^, primary human saphenous vein endothelial cells^49^, and rat retinal pigment epithelial cells^50^. Thus, further investigation of the pathway that mediate Fbln5 action on β-cell proliferation is required. These effects of Fbln5 on β-cell functions and β-cell proliferation might be explained by the distinct proliferative and functional state of the β-cells. A previous study showed that a high rate of insulin production suppressed β-cell proliferation because of increased ER stress, in a cell-autonomous manner^51^. On the other hand, genes involved in β-cell functions were suppressed when proliferation-related genes were upregulated in replicating β-cells^52^.

There is a report in the literature which suggests that another matricellular protein, SPARC, which is expressed in stromal cells within the islets, can regulate β-cell growth and survival by inhibiting growth factor responses^53^. Thus, the interactions between Fbln5 and pancreatic β-cell functions, which are still poorly understood, may represent novel molecular mechanisms involved in glucose metabolism and provide new insights for the treatment in diabetes.

In summary, we demonstrated that expression of the matricellular protein Fbln5 is upregulated by high ambient glucose concentrations in the pancreatic islets though glucokinase-dependent glucose and downstream Ca^2+^/calcineurin/NFAT signaling. Further study of the regulation of islet Fbln5 expression is warranted, especially in relation to glucose signaling and proliferation of β-cells.

## Methods

### Animals and Animal Care

All the animal procedures were performed in accordance with the guidelines of the Animal Care Committee of Yokohama City University. The protocol was approved by the Yokohama City University Institutional Animal Care and Use Committee (IACUC) (Permit Number: F-A-16-026). C57BL/6 J mice were purchased from Jackson. We backcrossed Fbln5 knockout (*Fbln5* ^−/−^) mice^16, 19^ with C57BL/6 J mice more than 10 times. Both *Fbln5* ^−/−^ mice and wild-type littermates were fed a standard chow (MF, Oriental Yeast, Tokyo, Japan) or a high-fat diet (Clea Japan, Tokyo, Japan). All the experiments were conducted on male littermates. Animal housing rooms were maintained at a constant room temperature (25 °C) and a 12-hour light (7:00 a.m.) /dark (7:00 p.m.) cycle.

### Adenovirus

*Fbln5*-overexpressing recombinant adenovirus (Ad-*Fbln5*)^18^ and GFP-expressing control adenovirus (Ad-GFP) were used for the experiments at a multiplicity of infection of 50 viruses per cell. In brief, the FLAG-tagged full-length rat Fbln5 was inserted in an adenoviral vector (pACCMVpLpA(−) loxP-SSP). Viruses were generated by transfection into the Human Embryo Kidney 293 (HEK293) cell line.

### Islet isolation and culture

Isolation of islets from mice was conducted using collagenase, as described in a previous report^54^. The isolated islets were cultured in RPMI 1640 medium (Wako Pure Chemical Industries) containing 5.6 mmol/L glucose supplemented with 10% FCS, 100 units/mL of penicillin, and 100 μg/mL of streptomycin. The islets were treated with 30 μmol/L of GKA Cpd A, 50 μmol/L of nifedipine, 10 μmol/L of FK506, 10 μmol/L of D-mannoheptulose (Toronto Research Chemicals), 200 μmol/L of diazoxide (Wako Pure Chemical Industries), 200 nmol/L of OSI-906 (Selleck Chemicals). All the reagents were added concomitantly to the medium in each experiment.

### Oral glucose tolerance test

All the mice were denied access to food for 14–16 hours before the oral glucose tolerance test (OGTT) and then orally loaded with glucose at 1.5 mg/g body weight. Blood glucose levels and serum insulin levels were determined using Glutest Neo Super (Sanwa Chemical Co. Kanagawa, Japan) and an insulin ELISA kit (Morinaga Institute of Biological Science, Yokohama, Japan), respectively.

### Glucose-stimulated insulin secretion in isolated islets and INS-1 cells

Ten islets isolated from *Fbln5* ^−/−^ mice and wild-type mice were incubated at 37 °C for 1.5 hours in Krebs-Ringer bicarbonate buffer containing 2.8, 11.1 or 22.2 mmol/L of glucose. When examining the effect of *Fbln5* deficiency on GKA-induced insulin secretion, islets were incubated at 37 °C for 1.5 hours in Krebs-Ringer bicarbonate buffer containing 2.8 mmol/L glucose with or without 30 µmol/L of GKA CpdA, or 11.1 mmol/L glucose without GKA CpdA. For measuring insulin content, islets were extracted with acid ethanol. INS-1 cells were infected with adenovirus (Ad-GFP or Ad-*Fbln5*) and cultured for 48 hours. Subsequently, the cells were incubated at 37 °C for 2 hours in Krebs-Ringer bicarbonate buffer containing 2.8, 11.1 or 22.2 mmol/L of glucose. Then, the insulin concentration in the assay buffer and insulin content was measured with an insulin ELISA kit.

### Cell culture

INS-1 (832/13) cells^55^ were cultured in RPMI 1640 containing 10 mmol/L of HEPES, 11.1 mmol/L of glucose, 10% FBS, 1 mmol/L of sodium pyruvate, 2 mmol/L of L-glutamine, 50 μmol/L of 2-mercaptoethanol, 100 units/mL of penicillin and 100 μg/mL of streptomycin. The cells were maintained at 37 °C in humidified air containing 5% CO_2_. Before the experiments, the INS-1 cells were starved by incubation in RPMI1640 medium containing 2.8 mmol/L of glucose, 100 units/mL of penicillin, and 100 μg/mL of streptomycin for 16 hours.

### Histological analysis

Pancreatic tissue sections from embryonic day15 and 8-week-old *Fbln5* ^−/−^ mice and wild-type mice were analyzed after formalin fixation and paraffin embedding. For non-paraffinized tissue staining, isolated islets from 8-week-old wild-type mice attached to 0.1%-gelatin-coated coverslips (Falcon) were analyzed after fixation with paraformaldehyde. Pancreatic islets isolated from 8-week-old wild-type mice were analyzed after fixation without paraffin embedding. The sections or attached islets on coverslips were immunostained with antibody directed against insulin (Santa Cruz Biotechnology), glucagon (Abcam), somatostatin (GeneTex), CD34 (Santa Cruz Biotechnology), or rabbit polyclonal anti-fibulin-5 (BSYN 1923; 1:100)^16^. FBLN5 signal was enhanced by tyramide signal amplification, using a TSA Fluorescein System (Perkin Elmer, NEL741001KT), in paraffin-embedding sections. Biotinylated secondary antibodies, a VECTASTAIN Elite ABC Kit, and a DAB Substrate Kit (Vector Laboratories) were used to examine the sections using bright-field microscopy to determine the β-cell mass, and Alexa Fluor 488-, 555- and 647-conjugated secondary antibodies (Invitrogen) were used for the fluorescence microscopy. Images were acquired using a BZ-9000 microscope (Keyence) or the FluoView FV1000-D confocal laser scanning microscope (Olympus). The proportion of the area of the pancreatic tissue occupied by β-cells was calculated using BIOREVO software (Keyence), as described previously^56^. The fluorescence levels of insulin in GKA-treated wild-type and *Fbln5* ^−/−^ islets were determined using Image J software. All images, which were acquired under the same condition, were converted to gray scale. Then, we randomly selected 5 regions of separate islets in each group and measured fluorescence levels. The fluorescent intensity were normalized by the mean background fluorescence levels.

### Proliferation Assay

Isolated islets from *Fbln5* ^−/−^ mice and wild-type mice were incubated with a modified thymidine analog, EdU (5-ethynyl-2’-deoxyuridine; Click-iT EdU; Invitrogen Cat. No. C10637) in the presence or absence of 30 μmol/L of GKA. After the treatment for 48 hours, the islets were fixed and sections were prepared of the embedded islets in 1% agarose-gel.

INS-1 cells were infected with an adenovirus vector (Ad-GFP or Ad-*Fbln5*) and cultured for 48 hours. Then, the cells were incubated with 10 µM of EdU for 3 hours and fixed. EdU incorporation and detection were performed as described in the manufacturer’s protocol. The images were taken using the FluoView FV1000-D confocal laser scanning microscope. We counted EdU-positive proliferative cells after adjusting the gain of fluorescence. At that condition there were no significant changes in the fluorescent intensity between GFP-positive cells and GFP-negative cells.

### Real-time PCR

Total RNA was isolated from the pancreatic islets using an RNase-free DNase and RNeasy Kit (Qiagen, Valencia, CA). cDNA was prepared using High Capacity cDNA Reverse-Transcription Kits (Applied Biosystems). Quantitative PCR was performed by using TaqMan Gene Expression Assays (7900 Real-Time PCR System; Applied Biosystems) with the THUNDERBIRD qPCR Master Mix (TOYOBO). All the probes were purchased from Applied Biosystems (mouse *Fbln5*; Mm00488601_m1, mouse *β-actin*; Mm00607939_s1, mouse *Ki67*; Mm01278617_m1, rat *Fbln5*; Rn0069712_m1, rat *β-actin*; Rn00667869_m1, rat *Ki67*; Rn01451446_m1). Data were normalized to the expression level of *β-actin*.

### Immunoblotting

For immunoblotting, isolated mouse islets and INS-1 cells were lysed in ice-cold RIPA buffer with protease and phosphatase inhibitor cocktail. The islets and cell extracts were subjected to immunoblotting. The primary antibodies used were rabbit anti-FBLN5 (BSYN1923) at the dilution of 1:100, or Anti-Fibulin-5-Antibody (Millipore) at the dilution of 1:5000, and β-actin (Sigma-Aldrich). Densitometry was performed using Image J software.

### Statistical analysis

All the data are expressed as the means ± SEM, and were analyzed using the Student’s t test or ANOVA. Differences between two groups were analyzed by Student’s t test (Figs 1a, 3b-c and e, 4, Supplementary Fig. S1b-c). For comparisons among more than two groups, we used the one-way ANOVA followed by the Tukey HSD post hoc test (Figs 1b ﻿and d–i, 2a and d, 3g, Supplementary Fig. S1a). When the data had unequal variance, we used Welch’s one-way ANOVA followed by the Games-Howell post hoc test (Fig. 2b,c,e,f). Differences were considered significant if the *p* value was <0.05 (*) or <0.01 (**).

## Electronic supplementary material

Supplementary FIgures S1-S4
