# Supplementary material for: Identification of the matricellular protein Fibulin-5 as a target molecule of glucokinase-mediated calcineurin/NFAT signaling in pancreatic islets
Source: Sci Rep. 2017 May 24;7:2364. doi: 10.1038/s41598-017-02535-0 (PMC5443834; doi:10.1038/s41598-017-02535-0)
Supplement: Supplementary file 1 — Supplementary FIgures S1-S4 [file 41598_2017_2535_MOESM1_ESM.pdf]

Supplementary Information:

Supplementary Figures S1-S4

Identification of the matricellular protein Fibulin-5 as a target molecule of glucokinase-mediated calcineurin/NFAT signaling in pancreatic islets

Tomoko Okuyama, Jun Shirakawa, Hiromi Yanagisawa, Mayu Kyohara, Shunsuke Yamazaki, Kazuki Tajima, Yu Togashi, and Yasuo Terauchi

Supplementary Fig. S1

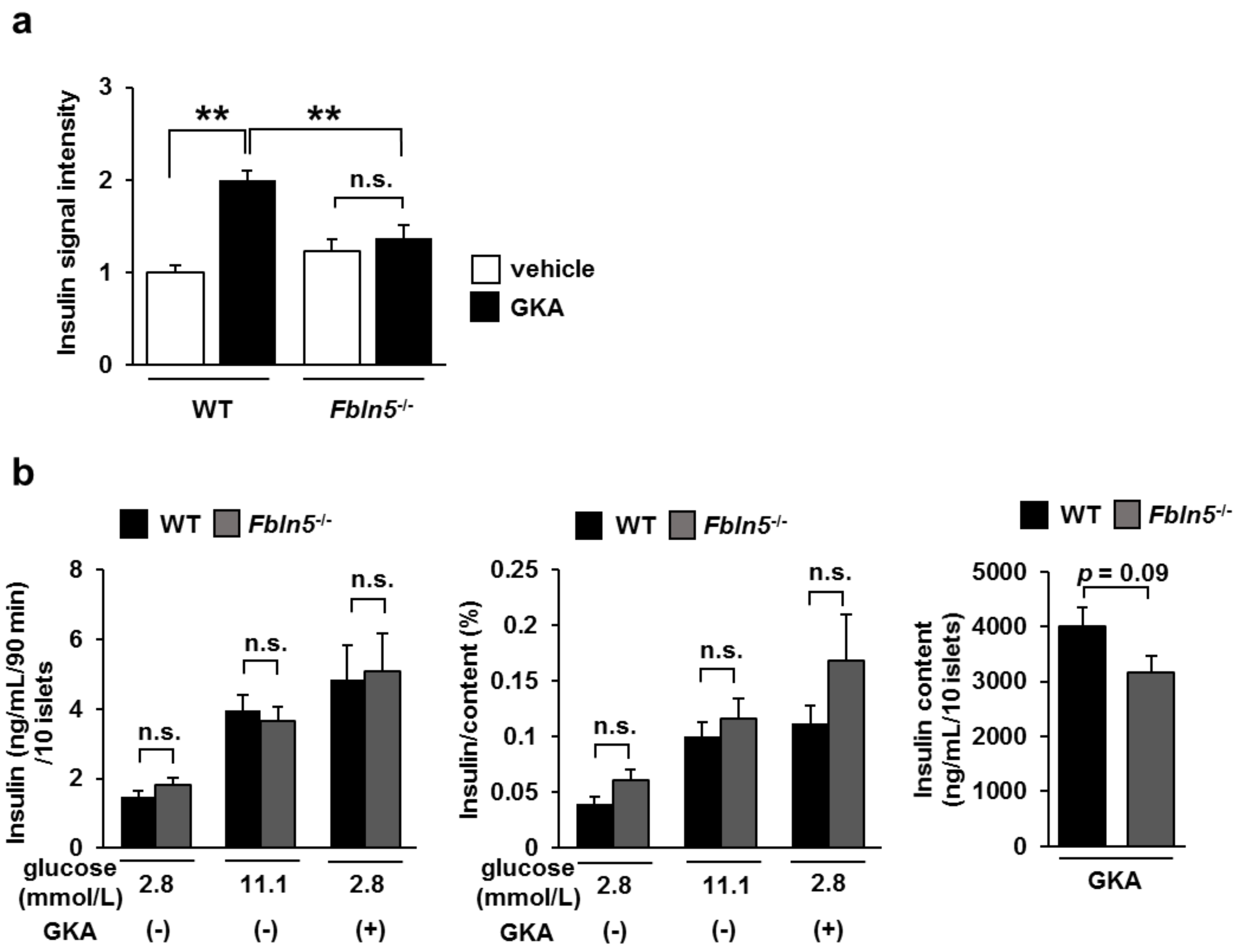

Supplementary Fig. S1. Normal GKA-stimulated insulin secretion in *Fbln5$^{-/-}$* islets.
(**a**) The fluorescent insulin signal intensity in GKA- and vehicle-treated islets isolated from 8-week-old wild-type and *Fbln5$^{-/-}$* mice was determined by using Image J. The intensity was measured in more than 5 sections of islets for each group. (**b**) GKA- or glucose-induced insulin secretion (*left*), insulin secretion rate corrected by the insulin content (*middle*), and insulin content after incubation with GKA and 2.8 mmol/L glucose for 1.5 hours (*right*) in ten isolated islets from 17-week-old *Fbln5$^{-/-}$* mice and wild-type mice (n = 9-16). Data are represented as means ± SEM. $^{**}p < 0.01$.

Supplementary Fig. S2

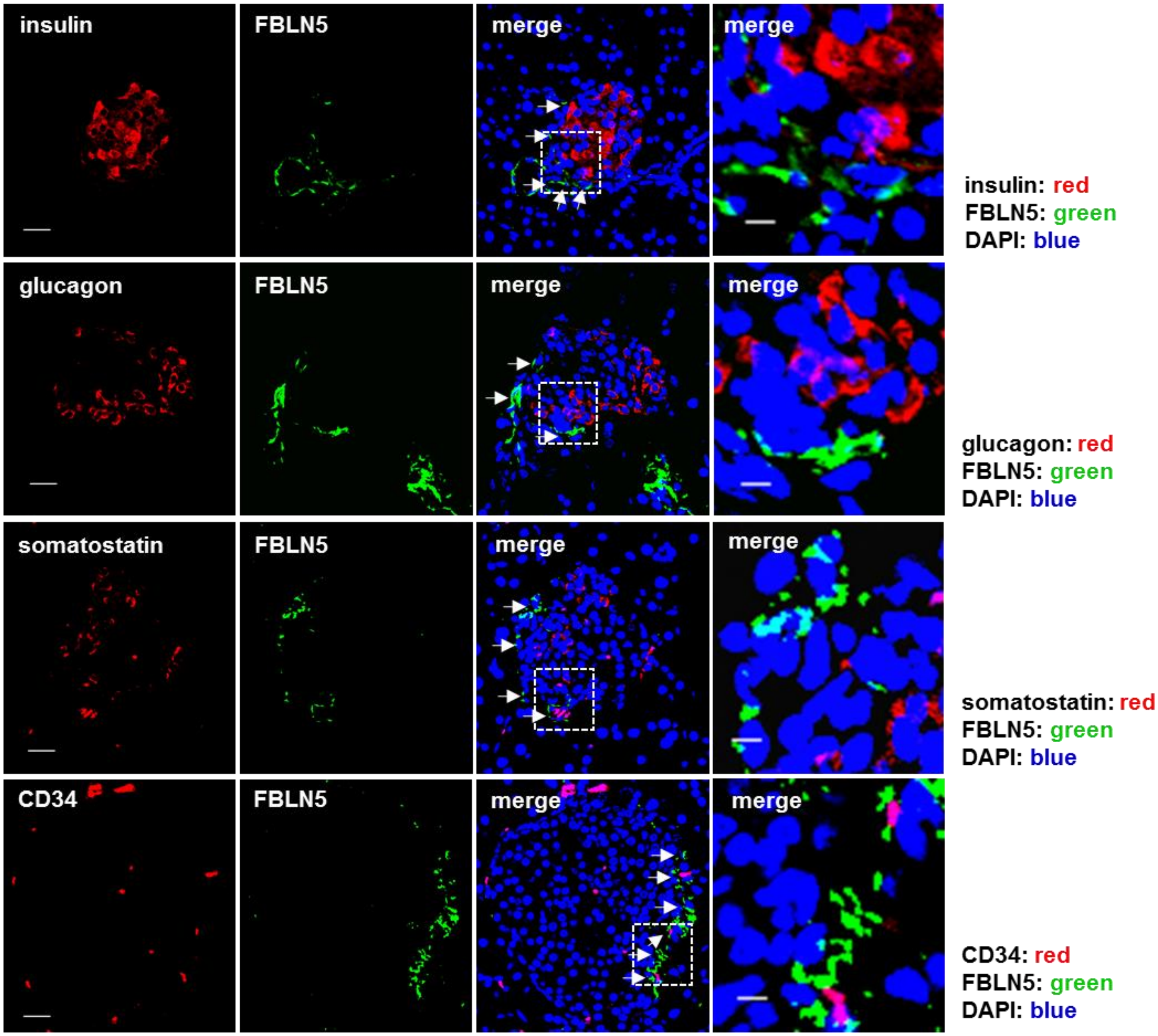

Supplementary Fig. S2. Immunohistochemical analysis for FBLN5 in paraffin-embedded pancreas from adult wild-type mice.

Representative paraffin-embedded pancreatic sections from 8-week-old wild-type mice stained with antibodies for FBLN5 (green), insulin (red), glucagon (red), somatostatin (red), and CD34 (red) are shown. The signal of FBLN5 is amplified with tyramide signal. Nuclei are stained blue with DAPI. The right panels are enlarged image of white dotted square. The scale bar represents 5 µm in the right enlarged panel and 20 µm in other panels. The white arrows indicate FBLN5-positive tissue in the islets.

Supplementary Fig. S3

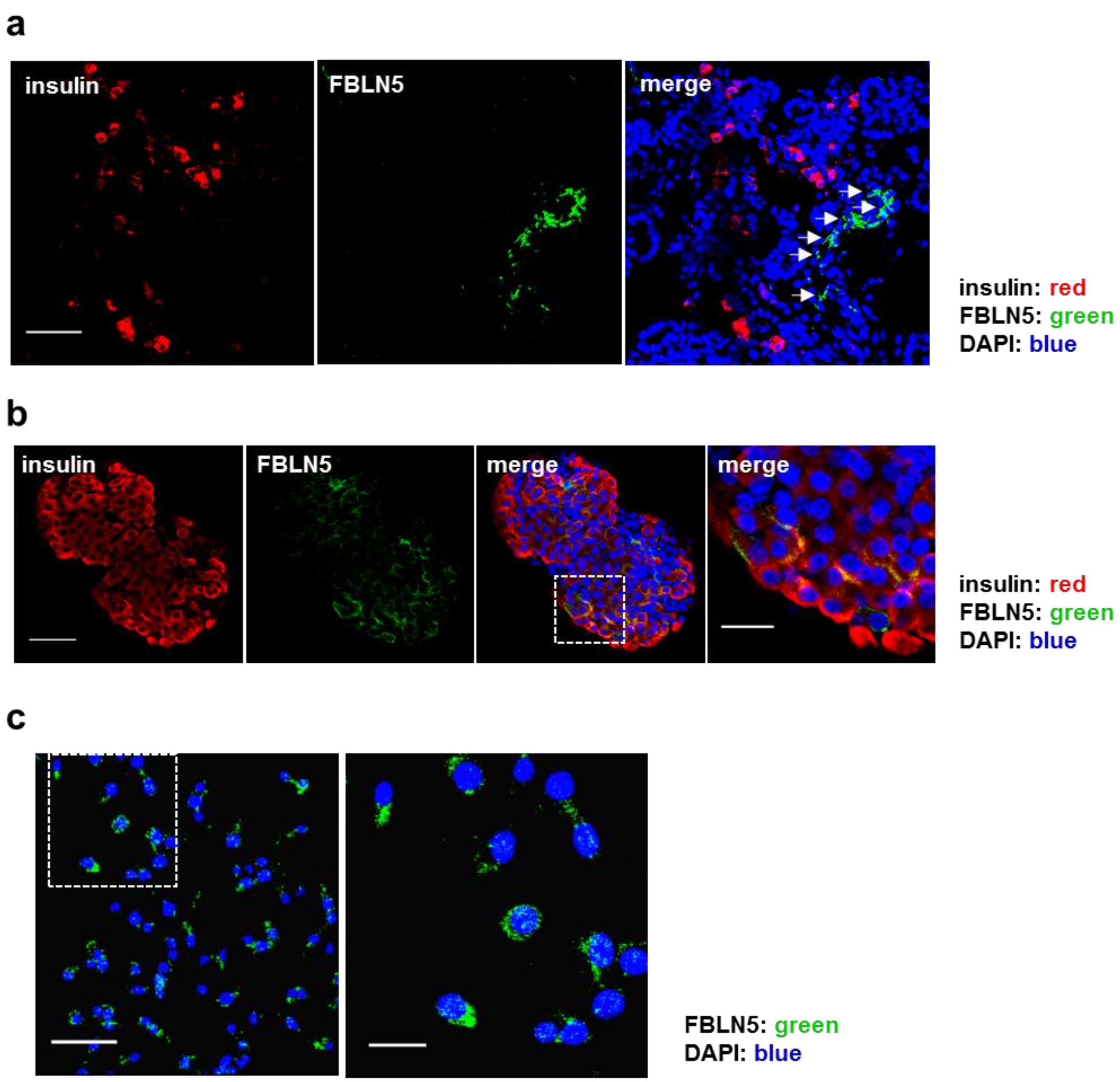

Supplementary Fig. S3. Immunohistochemical analysis for FBLN5 in fetal pancreas, non-paraffinized adult islets, or INS-1 cells.
(**a**) Representative paraffin-embedded pancreatic sections from fetal C57BL/6J mice at the embryonic day 15 stained with antibodies for insulin (red) and FBLN5 (green) are shown. The signal of FBLN5 is amplified with tyramide signal. Nuclei are stained blue with DAPI. The scale bar represents 50 µm. The white arrows indicate FBLN5-positive tissue. (**b**) The non-paraffinized islets isolated from 8-week-old C57BL/6J mice were stained with antibodies for insulin (red) and FBLN5 (green). Nuclei are stained blue with DAPI. The scale bar represents 20 µm in the right panel and 50 µm in other panels. (**c**) Representative image of INS-1 cells stained with FBLN5 (green). Nuclei are stained blue with DAPI. The scale bar represents 50 µm in the left panel and 20 µm in the right panel.

Supplementary Fig. S4

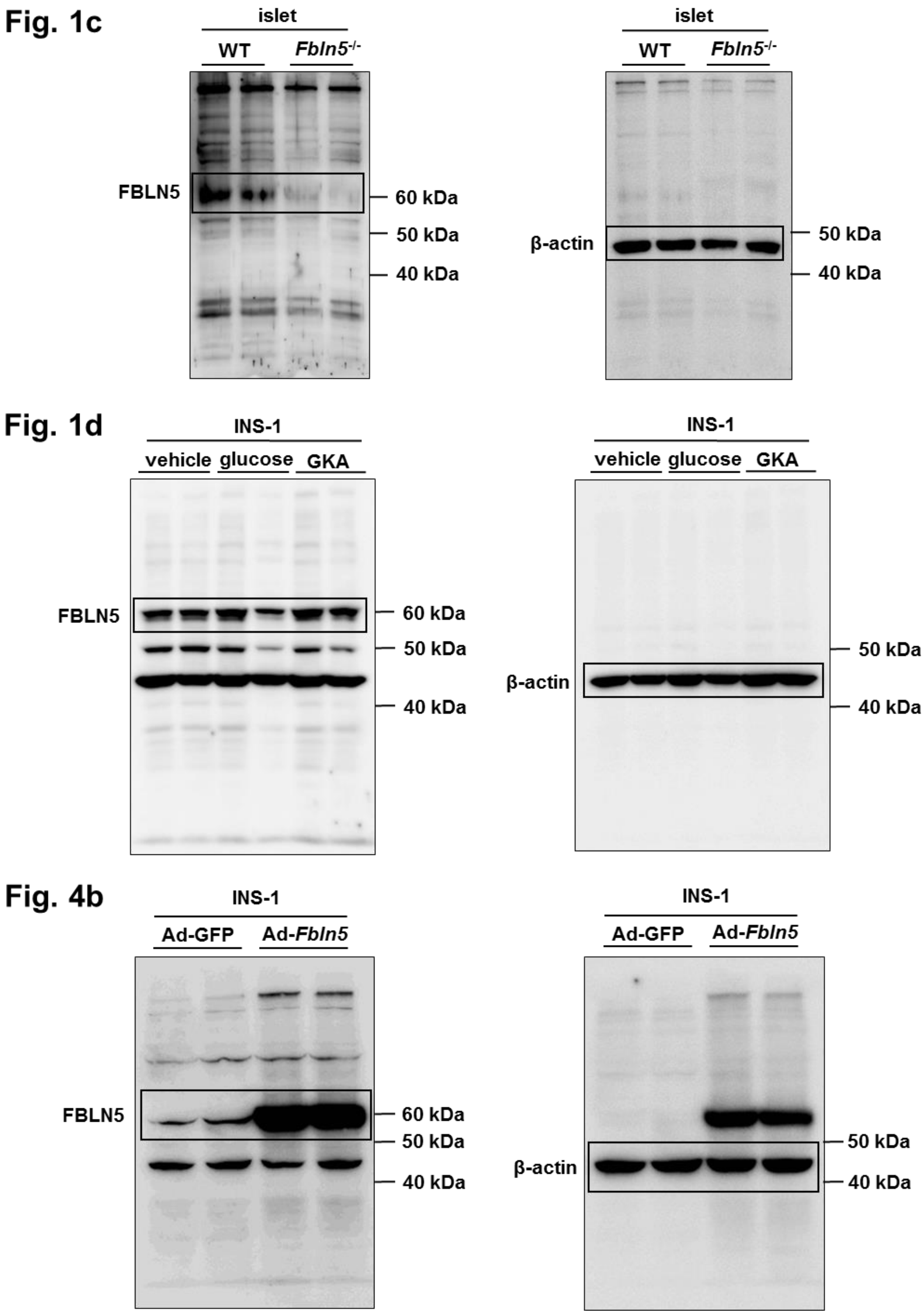

Supplementary Fig. S4. Uncropped images of key panels in main figures.
Black boxes indicate the cropped portion of each immunoblot presented in the corresponding main figure.
